# The Influence of Health Beliefs, of Resources, of Vaccination History, and of Health Anxiety on Intention to Accept COVID-19 Vaccination

**DOI:** 10.3389/fpsyg.2021.729803

**Published:** 2021-09-14

**Authors:** Adelina Mihaela Ştefănuţ, Mona Vintilă, Mihaela Tomiţă, Eugenia Treglia, Monica Alina Lungu, Rosella Tomassoni

**Affiliations:** ^1^Department of Psychology, Faculty of Sociology and Psychology, West University of Timişoara, Timişoara, Romania; ^2^Department of Social Work, Faculty of Sociology and Psychology, West University of Timişoara, Timişoara, Romania; ^3^Department of Human Sciences, Social and Health, University of Cassino, Cassino, Italy

**Keywords:** vaccination intention, COVID-19, Health Belief Model, resources, vaccination history

## Abstract

The aim of this study is to investigate whether Health Belief Model constructs, personal resources, vaccination history and health anxiety exert an influence on vaccination intention. To achieve this end, we carried out a cross-sectional study of 432 people drawn from a convenience sample who answered an online questionnaire. Multiple logistical regressions showed that perception of the severity of the disease, of the benefits of being vaccinated, of barriers, and of cues to action, along with the freeness and accessibility of the vaccine and general vaccination history, are significant predictors regarding vaccination intention.

## Introduction

According to the World Health Organization (WHO), the first cases of infection with the new coronavirus (hereafter called SARS-COV-2) appeared in December 2019 in Wuhan, China in the form of a “viral pneumonia.” The rapid spread of this infection led the WHO to declare a public health state of emergency on 30 January 2020. At the time of writing, COVID-19 has affected over 123 million people worldwide and has caused the death of over 2.7 million (World Health Organization, 2021a). At the time of the study, Romania faced the 3rd wave of the pandemic, with the number of daily active cases decreasing after the peak recorded on November 16 of 59,623 active cases (World Health Organization, 2021b) and the number of intensive care beds occupied almost at full capacity. At the end of December, the total number of infections since the beginning of the pandemic was 632,263 and the total number of deaths was 17,722 (Worldometer, 2021), Romania having a resident population of 19.3 million (National Institute of Statistics, 2021).

In the absence of any specific treatment, efforts to limit and halt the spread of the infection have been focused on encouraging the adoption of preventive behaviors and on the development of a vaccine. To this end, the governments of different countries imposed unprecedented public health measures, including quarantining those returning from abroad, closing non-essential services, closing schools, and requiring people to work from home wherever possible. However, these measures have had a negative impact in psychological (Brooks et al., 2020), social (Chen et al., 2020), and economic (Nicola et al., 2020) terms. This being the case, the development of an effective vaccine became even more important, with universities, pharmaceutical companies, and firms specializing in biotechnology all becoming involved in the research process. The end of 2020 saw a number of different vaccines against COVID-19 coming onstream and the start of a vaccination campaign. To date, over 3.79 billion doses have already been administered (Our World in Data, 2021a).

The vaccination campaign started in Romania on December 27. According to the national vaccination strategy, the campaign included three stages: in the first stage, vaccination of health and social services workers was planned, in the second stage, vaccination of the population at high risk of developing severe forms of the disease and workers in the essential fields was planned, and the last stage was addressed to the general population (Romanian Government, 2021). To date, 24.52% of the Romanian population was fully vaccinated and 0.73% of it was only partially vaccinated (Our World in Data, 2021b).

While vaccination is of paramount importance for stopping the pandemic (Kennedy et al., 2011), since it is the most successful and most effective means of preventing infectious diseases, the experience of the past shows that it is not enough for a vaccine to exist; it also needs to be accepted by the population. In earlier pandemics, vaccine acceptance differed greatly, with a figure of 17%, for example, being recorded in a study carried out in France (Schwarzinger et al., 2010) and one of 67% in a study in Australia (Eastwood et al., 2010).

For the SARS-COV-2 virus, herd immunity can be attained only if at least 67% of the population are vaccinated (the calculation assumed an R value of three) (Kwok et al., 2020). Research has demonstrated that levels of COVID-19 vaccine acceptance differ extremely widely: one study carried out in China found that 91.3% of participants were prepared to be vaccinated (Wang et al., 2020), yet another, from the US, reported that 68% of its participants were in favor of the idea of being vaccinated (Pogue et al., 2020). In addition, previous experience demonstrates that we also need to take into account the fact that there is a difference between accepting the vaccine in principle and actual vaccination behavior, the latter running at a lower percentage (Raude et al., 2010).

Given the fact that there is currently no specific treatment for COVID-19 and that preventive measures are not without their negative consequences on many levels, it is extremely important that we should understand the factors that influence people's attitudes toward being vaccinated. Previous studies have shown that vaccine acceptance is a decision that is both complex and context-specific (Larson et al., 2014), influenced by such factors as perception of the risk of the disease and of the seriousness of the consequences of contracting it, perception of the safety of the vaccine and of its effectiveness, vaccination history, and a range of socio-demographic characteristics (being elderly, being male, belonging to an ethnic minority, being a health service professional) (Bish et al., 2011; Nguyen et al., 2011). In the specific context of COVID-19, research has demonstrated a link between vaccination intention and the belief in conspiracy theories (Earnshaw et al., 2020), confidence in the government and in those who developed the vaccine (Freeman et al., 2020), low economic status and limited education (Bertoncello et al., 2020), the effectiveness of the vaccine (Pogue et al., 2020; Wang et al., 2020), side effects of the vaccine and how long it has been tested for (Pogue et al., 2020), being married or unmarried, and cost (Wang et al., 2020). While there are some studies that have analyzed the influence of a range of factors on the acceptability of COVID-19 vaccination, few of these have a sound theoretical basis. Our research, which is based on the Health Belief Model and on resource theory, is designed to investigate the impact of beliefs about health, resources, vaccination history, and health anxiety on COVID-19 vaccination intention.

The Health Belief Model (HBM) (Rosenstock, 1974) comprises the following constructs: *perceived susceptibility* (this refers to the probability that the person will contract the disease), *perceived severity* (this takes into account the seriousness of the consequences of becoming ill), *perceived benefits* (the positive consequences of adopting preventive behaviors), *perceived barriers* (obstacles that could prevent the person adopting the intended behavior), and *cues to action* (stimuli that contribute to the decision to adopt the intended behavior). The model has been used to account for a variety of health-related behaviors including vaccination (Cheney and John, 2013; Mo and Lau, 2015). Regarding the COVID-19 vaccination, one study has shown that it is perception of the benefits of vaccination COVID-19 that has the greatest influence on the development of a firm intention to be vaccinated (Wong et al., 2020), while another has demonstrated that perceptions of susceptibility and of the seriousness of COVID-19 strongly influence a desire to be vaccinated against it (Graffigna et al., 2020).

Since the HBM does not take account of contextual factors which might be involved in a decision regarding vaccination, the next set of factors we analyzed from the perspective of their potential influence on vaccination intention have to do with the resources that a person can access as a response to COVID-19. In the present research we find, under the general heading of resource theory, both micro-level and macro-level resources. That is, the scope of the investigation extends both to individual resources (educational level, knowledge regarding the disease and its prevention, personal experience of the disease, experience of the disease among the person's social circle) and to interpersonal resources (available social support) and resources available in society (the fact that the vaccine is free, the fact that the vaccination procedure is accessible).

There are previous studies which showed that people's attitude related to health and for maintaining health can differ a lot according in different countries, according to the solutions policymakers found in order to implement a healthy lifestyle (Vintilă et al., 2009). Rural environment and old age can be sources of disadvantage, if the family system and the social support are not appropriate (Eglite et al., 2009). Neighbors, the larger family system and the partner can be significant resources that can help people understand the seriousness of this disease and the necessity to accept vaccination due to the lack of an efficient treatment until now (Vintilă et al., 2019). The dyad patient-partner/caregiver has been proven to react similarly in cases of serious diseases, ex. cancer, this is why many recent studies have gone form analyzing just the patient to analyzing the dyad (Ştefănuţ and Vintilă, 2019).

Previous literature in the field has highlighted the fact that personal vaccination history influences the acceptability of a new vaccine (Bish et al., 2011; Nguyen et al., 2011), an influence that would appear to hold true for the COVID-19 vaccine as well (Graffigna et al., 2020; Wang et al., 2020). One aim of our study is to discover whether this result can be confirmed for the Romanian population too.

Previous studies suggest a link between a diagnosis of mental disturbance and a low probability that a person will access prevention services, vaccination included (Lord et al., 2010). Health anxiety, however, is characterized by a person having an excessive preoccupation with investigating the state of their health and a conviction that they are suffering from or are going to suffer from a serious disease that is as yet undiagnosed. Consequently, in the context of the pandemic, bringing as it does the possibility of contracting a virus that may result in developing a serious form of the disease or even dying, a question arises as to whether the association that exists between health anxiety and vaccination intention is different from the negative one (already highlighted in the literature) between mental disturbances in general and vaccination intention.

Summarizing, in contrast to the Health Belief Model which focuses on the role that personal beliefs associated with the disease have in determining behaviors that promote health, resource theory highlights individual or existing assets at the community level that can be used by people in response to the threat of disease. On the other hand, studies have highlighted the influence of past behaviors, the results of which are known and which can function as reinforcements (vaccination history) on the acceptability of a new vaccine, as well as the association between mental disturbance and access to vaccination as a preventive behavior. Thus, in order to identify the aspects that should be considered in the vaccination promotion strategies for COVID-19, the authors wanted to analyze the influence of these groups of cognitive, contextual, behavioral and mental health factors on the same population sample. Bearing in mind what has been explained above, the specific research hypotheses of the current study are as follows:

H1: personal vaccination history has a significant influence on COVID-19 vaccination intention.
H2: personal resources, social support and resources available in society have a significant influence on COVID-19 vaccination intention.
H3: HBM constructs (susceptibility, severity, benefits, barriers, and cues to action) have a significant influence on COVID-19 vaccination intention.
H4: health anxiety has a significant influence on COVID-19 vaccination intention.

The novel elements of this research are concerned with its investigation of the influence of resources and of health anxiety on the acceptability of COVID-19 vaccination. A further aim of the study is to provide additional information to supplement that already available in the literature with regard to the way in which HBM constructs (susceptibility, severity, benefits, barriers, and cues to action) and vaccination history influence this acceptability in the context of the COVID-19 pandemic. Knowing the factors involved in people's decision to be (or not to be) vaccinated will make it possible to pinpoint vaccination promotion strategies that target different groups within the population. A non-homogeneous approach of this kind is regarded as holding out greater chances of success than an undifferentiated one (French et al., 2020).

## Methods

### Study Design

A cross-sectional design of study was employed to evaluate the influence of personal vaccination history, resources, HBM constructs and health anxiety on the decision to be vaccinated.

### Procedure and Participants

This online research was carried out in the West University, Timişoara, Romania in December 2020. To recruit participants in this study we used exponentially non-discriminatory snowball sampling, a non-probability sampling technique. According to this technique, a series of participants are recruited, then they recommend other people willing to participate in the research and the process can continue. In the case of our study, the first level of recruited participants consisted of students from the Faculty of Sociology and Psychology at the West University of Timisoara. The second level of participants consisted of people invited by students (friends, relatives) to participate in research.

Students in the Sociology and Psychology Faculty were informed about the opportunity to participate, along with their invitees, in this piece of online research, provided they fulfilled the criteria of being at least 18, of knowing Romanian and of having Internet access. Bonus marks were awarded in the examinations as a means of encouraging participation. Those who expressed an interest in taking part in the research were given access to an online form so that they could answer the questionnaires. This made it possible to gather demographic data, vaccination history data, and information about resources, and participants' beliefs about health and level of health anxiety were evaluated.

This study was approved by the Ethics Committee of West University of Timişoara (decision no. 5224/ 03.02.2021). All participants expressed informed consent.

The data that support the findings of this study are openly available in osf.io repository.

### Variables and Instruments

Intention to be vaccinated against COVID-19 was the dependent variable investigated, and vaccination history, resources, HBM constructs, and health anxiety were independent variables. Demographic data was also collected.

#### Demographic Data

The questionnaire concerned with demographic data consisted of questions about age, gender, relationship status (single/in a relationship), living environment (urban/rural), occupational status (professionally active/inactive), and the existence of comorbidities (with/without relevant comorbidities).

#### Vaccination History

Vaccination history was evaluated with the help of two questions (Wang et al., 2020). The first was concerned with whether or not the person had had the flu vaccine in the previous flu season, while the second asked in general terms about acceptance of vaccines previously suggested.

#### Resources

The resources taken into consideration in this study were level of education, knowledge about the disease and its prevention, social support available, the accessibility of the vaccine and experience of the pandemic. Knowledge about COVID-19 and its prevention was evaluated by means of seven items developed by authors specifically for this research. The items concerned with social support asked about numbers of good interpersonal relationships and whether the person had people they could turn to for support when in need (Kim and Kim, 2020). The section dealing with the impact of vaccine accessibility on vaccination intention had one item concerned with the fact that the vaccine being free might encourage the person to decide to be vaccinated and another that took account of the fact that the accessibility of the vaccination procedure (method, frequency, distance from home to the vaccination location) might have an important influence on the decision to be vaccinated (Wang et al., 2020). Experience of the pandemic was evaluated with the help of two questions. The first asked about personal experience of COVID-19 and the second question dealt with experience of the disease among the person's social circle. These questions were developed by the authors specifically for this research. The construct validity of the scales used to evaluate the resources was ensured by debating the items used within the team of specialists.

#### HBM Constructs

The discovery that there was no standardized questionnaire for evaluating HBM constructs in the context of the COVID-19 pandemic while taking account of vaccination intention led to our devising such a questionnaire for use in the study. For the susceptibility and severity constructs, the authors used items they had used in an earlier study dealing with COVID-19 prevention behaviors (Ştefănuţ and Vintilă, submitted)^1^. After reviewing the literature, they supplemented these with items that focused on the benefits, barriers, and cues to action constructs. The resulting questionnaire was evaluated and debated by a team of mental health specialists. Of the items proposed, 19 were judged to be relevant and found their way into the final version of the questionnaire. In this way we decided upon five items each that dealt with susceptibility and severity and three items each for benefits of vaccination, possible barriers standing in the way of vaccination, and cues to action. Responses were evaluated on a 5-point Likert scale (1—totally disagree, 2—disagree, 3—neither agree nor disagree, 4—agree, and 5—strongly agree). The total score for each construct was calculated by summing points awarded for the items belonging to that construct. The Exploratory Factor Analysis applied for this new questionnaire showed that as we expected, the 19 items loaded significantly in five factors explaining 54.45% of the variance. One of the susceptibility items loaded approximately equally in the susceptibility factor and in the severity factor but as a result of the content analysis (“*I believe I will contract this virus*”). It was finally kept in the susceptibility factor. Values obtained for the α Cronbach coefficient were as follows: for susceptibility 0.70, for severity 0.75, for benefits 0.88, for barriers 0.89, and for cues to action 0.77.

#### Health Anxiety

This was evaluated with the aid of the Health Anxiety Inventory, the 18-item version (Salkovskis et al., 2002). The items in this questionnaire are based on the cognitive theory of health anxiety and each has four possible responses, scored from 0 to 3 depending on the degree of salience of the thought concerned. One sample item reads as follows: *I never think I have a serious illness/I sometimes think I have a serious illness/I often think I have a serious illness/I usually think that I am seriously ill*. The total score is obtained by summing the points. Internal consistency for the sample used in this study was calculated to be 0.85.

#### Vaccination Intention

This was investigated by the use of the question “*Do you intend to be vaccinated against COVID-19 when a vaccine becomes available?*”

Table 1 reproduces the contents of the questionnaire used to evaluate vaccination history, resources, HBM constructs, and vaccination intention.

**Table 1:** Questionnaire items used to operationalise measurement of variables.

**Concept**	**Item**	**α Cronbach/ correlation**
**Vaccination history**		
Vaccination history	Did you have the flu vaccine last season? (yes/no)	0.20^a^
	In the past, have you accepted all the vaccines that were recommended to you? (yes/no)	
**Resources**		
Education	Highest level of education finished: medium (middle school, high school)/higher (undergraduate degree, master's, doctorate)	NA
Knowledge about COVID-19 and its	I know the main symptoms of the disease (fever, dry cough, respiratory difficulties, loss of the sense of smell or taste, headaches, digestive disturbances) (yes/no)	NA
prevention	I know which comorbidities combine with COVID-19 to cause a more severe form of the disease (cardiovascular conditions, chronic kidney diseases, chronic pulmonary diseases, chronic liver diseases, diabetes, cancer, neurological and neuromuscular conditions, obesity (yes/no)	
	I am familiar with the measures for preventing COVID-19 infection (wearing a mask, social distancing, frequent handwashing, avoiding touching the face, using a paper tissue when I sneeze or cough) (yes/no)	
	I know that supportive treatment helps the majority of patients to recover (yes/no)	
	I know that people who have been in contact with a person confirmed with coronavirus have to self-isolate (yes/no)	
	I know how many days a contact of a person confirmed with coronavirus has to self-isolate (14) (yes/no)	
	I know that vaccines to prevent COVID-19 have already been developed (yes/no)	
Available social support	I have good relationships with a large number of people (yes/no)	0.32^a^
	There are people who can help me when I am in difficulty (yes/no)	
Accessibility of the	The fact that the vaccine is free encourages me to be vaccinated (yes/no)	0.39^a^
vaccine	The accessibility of the vaccine (vaccination method, frequency, distance from my home to the vaccination center) is important in my decision about whether or not to be vaccinated (yes/no)	
Personal experience of the pandemic	What has been your experience of this disease? (I have not had this disease/I have had an asymptomatic form of the disease/I have had a form of this disease that required treatment)	NA
Experience of the pandemic among social circle	What has been the experience of this disease among your acquaintances? (I do not know anyone who has contracted the virus/I know people who have had an asymptomatic form of the disease/I know people who have had a form of this disease that required treatment/I know people who have died from this disease)	NA
**HBM constructs**		
Susceptibility	The nature of my daily activities means that I have a high probability of contracting the virus	0.70
	My health, through visits to the doctor and the treatments I need, makes me more exposed to the virus	
	The activities of my family members increase my chances of becoming infected with the virus	
	I am worried that I will contract this virus	
	I believe I will contract this virus	
Severity	The disease caused by this virus is more serious than other diseases	0.75
	The problems I would face as a result of this disease would last a long time	
	My education/professional activity would be seriously affected if were to have this disease	
	If I were to have this disease, my family's health would be in danger	
	The mortality rate from this disease is a high one	
Benefits	My being vaccinated will reduce the probability of getting COVID-19	0.88
	My being vaccinated will reduce the probability of my family members developing the disease	
	My vaccination will allow me to resume some of my pre-pandemic activities	
Barriers	I am worried about how effective the vaccine is	0.89
	I am worried about possible side effects of the vaccine	
	I am worried about whether or not the vaccine will be distributed and administered correctly	
Cues to action	I will be vaccinated if public figures I trust are vaccinated in public	0.77
	I will be vaccinated if my family doctor encourages me to do so	
	I will be vaccinated if I am given appropriate information about the vaccine	
**Vaccination intention**		
Vaccination intention	Do you intend to be vaccinated against COVID-19 when the vaccine becomes available? (definitely not/probably not/I have not yet made up my mind/probably/definitely)	NA

a *For this section which contains only two items, the correlation coefficient was calculated, obtaining a statistically significant correlation at the level of 0.01*.

### Statistical Analysis

The SPSS v20 program was used for statistical data analysis. Vaccination intention was analyzed both globally and in relation to different demographic characteristics, vaccination history, resources, HBM constructs, and health anxiety. In order to determine associations between the possible predictors selected and vaccination intention, the χ^2^ correlation coefficient was calculated. Those independent variables that were found to be statistically significantly associated with vaccination intention were noted so that their influence on it could be assessed. Since vaccination intention was operationalised as the categoric level variable, multinomial regression analysis was employed.

No preventive power analysis was performed. However, applying the formula *z*^2^ × *p* × (1–*p*)/*M*^2^ for which we considered a 95% confidence interval (*z*^2^ = 1.96), a statistical significance threshold of 0.05 (*M*^2^ = 0.0025) and an acceptance rate of 45% (*p* = 0.45) obtained for the present study, resulted in a number of 380 participants, so we can say that the sample size included in the study was adequate.

## Results

### Demographic Characteristics

There were 432 participants in the study, 175 women and 257 men. For female participants the minimum age was 18 and the maximum 55 (*m* = 22.71, *SD* = 8.00). For male participants the minimum age was 18 and the maximum 73 (*m* = 27.9, *SD* = 12.22). Most participants were under 35 years old (81.7%), 16.4% were between 35 and 55 years old and only 1.9% were over 55 years old. 59.7% of participants are in a relationship and 73.8% live in an urban environment. Regarding occupational status, 96.1% are professionally active; the great majority, 83.1%, have not been diagnosed with chronic conditions (Table 2).

**Table 2:** Sociodemographic characteristics and COVID-19 vaccination intention.

**Demographics**	**Total**	**Vaccination intention**				
	***N* (%)**					
		**Definitely not**	**Probably not**	**Undecided**	**Probably**	**Definitely**
		***N* (%)**	***N* (%)**	***N* (%)**	***N* (%)**	***N* (%)**
**Age**						
Age ≤ 35	353 (81.7)	39 (11)	50 (14.2)	116 (32.9)	92 (26.1)	56 (15.9)
35 < Age <55	71 (16.4)	7 (9.9)	2 (2.8)	20 (28.2)	23 (32.4)	19 (26.8)
55 ≤ Age	8 (1.9)	–	–	2 (25)	3 (27.5)	3 (27.5)
**Gender**						
Male	257 (59.5)	30 (11.7)	39 (15.2)	68 (26.5)	68 (26.5)	52 (20.2)
Female	175 (40.5)	16 (9.1)	13 (7.4)	70 (40)	50 (28.6)	26 (14.9)
**Relationship status**						
Single	174 (40.3)	19 (10.9)	18 (10.3)	59 (33.9)	49 (28.2)	29 (16.7)
In a relationship	258 (59.7)	27 (10.5)	34 (13.2)	79 (30.6)	69 (26.7)	49 (19.0)
**Living environment**						
Urban	319 (73.8)	30 (9.4)	41 (12.9)	92 (28.8)	97 (30.4)	59 (18.5)
Rural	113 (26.2)	16 (14.2)	11 (9.7)	46 (40.7)	21 (18.6)	19 (16.8)
**Occupational status**						
Professionally active	415 (96.1)	43 (10.4)	52 (12.5)	132 (31.8)	114 (27.5)	74 (17.8)
Professionally inactive	17 (3.9)	3 (17.6)	–	6 (35.3)	4 (23.5)	4 (23.5)
**Disease history**						
Chronic conditions—no	359 (83.1)	43 (12.0)	39 (10.9)	117 (32.6)	97 (27.0)	63 (17.5)
Chronic conditions—yes	73 (16.9)	3 (4.1)	13 (17.8)	21 (28.8)	21 (28.8)	15 (20.5)

### Vaccination History

Looking at vaccination history in general, 62% of participants said that they had accepted all the vaccines they had been advised to take in the past, while 38% had refused one or more vaccines. It was noteworthy that only 13% of participants had been vaccinated against influenza in the last flu season and this can be explained by the fact that many of the participants were young students, not worried about the flu.

### Resources

With regard to participants' personal resources, a large majority of them, 74.5%, had a medium level of education. The lowest questionnaire score for knowledge about COVID-19 and how to prevent it was 3 points and the highest was 7. Since both personal experience of the disease and the experience of participants' social circles were regarded as significant resources for understanding the context, these too were assessed. It was found that the great majority of respondents, 89.6%, had not been infected, 4.9% had had an asymptomatic form of the disease, and 5.6% had had a form of the disease that required treatment. In addition, 18.1% of participants did not know anyone who had had COVID-19, 27.5% had acquaintances who had had an asymptomatic form of the disease, 41.4% knew people who people who had needed treatment and 13% knew people who had died from the SARS-COV-2 virus.

Turning to interpersonal resources, it appears from their responses to the questions about social support that study participants do have support of this kind available. 98.6% of respondents said that there were people who could help them if they were in difficulty and 88% said that they had good relationships with a large number of people.

Looking at resources available in society, 52.8% of those who registered for the study agreed that the fact that the vaccine was free encouraged them to be vaccinated, while 55.8% thought that vaccine accessibility (method of vaccination, frequency, distance from their home to the vaccination center) was a major factor influencing their deciding to be vaccinated.

### Health Beliefs

42.88% of participants felt that they were very susceptible to becoming infected, while 44.67% thought that the consequences of the disease were severe. 44.21% of respondents perceived the benefits of vaccination as very great; 45.13% felt that the obstacles that might stand in the way of their deciding to be vaccinated were substantial. At the same time, 48.84% of subjects said that the cues to action listed in the questionnaire would represent a considerable encouragement to them to be vaccinated.

### Health Anxiety

In analyzing levels of health anxiety, a cut-off of 15 (Tang et al., 2007) was used. 11.34% of respondents scored between 15 and 17, which, while representing a very high level of health anxiety, falls short of the clinical threshold. Another 22.68% scored 18 or above, which is a clinical diagnosis.

### Vaccination Intention

18.06% of those participating in the study said that they were definitely going to be vaccinated and a further 27.3% that they would probably be vaccinated. 10.6% of participants maintained that they would definitely not be vaccinated and a further 12% that they would probably not be vaccinated. The striking feature, however, is the figure of 31.9% for people who had not yet made up their minds in this regard. Table 2 presents COVID-19 vaccination intention in relation to demographic characteristics.

We went on to analyze vaccination intention in relation to vaccination history, resources, beliefs connected with health, and health anxiety.

The χ^2^ correlation coefficient between possible predictors and the criterion (intention to be vaccinated) was calculated. Results are shown in Table 3. It may be seen that not all the variable selected as possible predictors show a significant correlation with the criterion. Statistically significant relationships were obtained between COVID-19 vaccination intention and history of flu vaccination in the previous season, vaccination history in general, the vaccine being free, the accessibility of the vaccine, people's social circle's experience of the disease, and perception of severity, benefits, barriers, and cues to action. In analyzing factors influencing vaccination intention we therefore took into account as predictor variables only those variables that displayed a significant correlation with vaccination intention.

**Table 3:** Association between possible predictors and vaccination intention.

	**Vaccination intention**					
	**Definitely not**	**No**	**Undecided**	**Yes**	**Definitely**	**χ^2^**
	***N* (%)^**a**^**	***N* (%)^**a**^**	***N* (%)^**a**^**	***N* (%)^**a**^**	***N* (%)^**a**^**	
	***m* (*SD*)^**b**^**	***m* (*SD*)^**b**^**	***m* (*SD*)^**b**^**	***m* (*SD*)^**b**^**	***m* (*SD*)^**b**^**	
**MODEL 1: PREDICTORS—VACCINATION HISTORY**						
**Vaccinated against flu in previous season**						
No	45 (12)	47 (12.5)	119 (31.6)	104 (27.7)	61 (16.2)	10.86^*^
Yes	1 (1.8)	5 (8.9)	19 (33.9)	14 (25)	17 (30.4)	
**Accepted all previous vaccinations**						
No	38 (23.2)	30 (18.3)	56 (34.1)	25 (15.2)	15 (9.1)	73.65^**^
Yes	8 (3)	22 (8.2)	82 (30.6)	93 (34.7)	63 (23.5)	
**MODEL 2: PREDICTORS—RESOURCES**						
**Education**						
Medium	36 (11.2)	41 (12.7)	106 (32.9)	87 (27)	52 (16.1)	3.8
Higher	10 (9.1)	11 (10)	32 (29.1)	31 (28.2)	26 (23.6)	
**Good relationships with many people**						
No	6 (11.5)	7 (13.5)	15 (28.8)	16 (30.8)	8 (15.4)	0.81
Yes	40 (10)	45 (11.8)	123 (32.4)	102 (26.8)	70 (18.4)	
**There are people who can help them**						
No	1 (16.7)	–	–	2 (33.3)	3 (50)	6.4
Yes	45 (10.6)	52 (12.2)	138 (32.4)	116 (27.2)	75 (17.6)	
**Is encouraged by the vaccine being free**						
No	45 (22.1)	43 (21.1)	86 (42.2)	25 (12.3)	5 (2.5)	170.35^**^
Yes	1 (4)	9 (3.9)	52 (22.8)	93 (40.8)	73 (32)	
**Is encouraged by the accessibility (proximity …) of the vaccine**						
No	40 (20.9)	34 (17.8)	49 (25.7)	38 (19.9)	30 (15.7)	55.71^**^
Yes	6 (2.5)	18 (7.5)	89 (36.9)	80 (33.2)	48 (19.9)	
**Personal experience of COVID-19**						
Has not been infected	42 (10.9)	48 (12.4)	125 (32.3)	106 (27.4)	66 (17.1)	
Has had an asymptomatic form of the disease	3 (14.3)	1 (4.8)	6 (28.6)	3 (14.3)	8 (38.1)	9.47
Has had a form of the disease which required treatment	1 (4.2)	3 (12.5)	7 (29.2)	9 (37.5)	4 (16.7)	
**Experience of COVID-19 among social circle**						
Does not know people who have been infected	13 (16.7)	5 (6.4)	28 (35.9)	20 (25.6)	12 (15.4)	
Knows people who have had an asymptomatic form	16 (13.4)	20 (16.8)	40 (33.6)	25 (21)	18 (15.1)	23.09^*^
Knows people who have required treatment	14 (7.8)	24 (13.4)	52 (29.1)	58 (32.4)	31 (17.3)	
Knows people who have died of COVID-19	3 (5.4)	3 (5.4)	18 (32.1)	15 (26.8)	17 (30.4)	
**Knowledge about COVID-19**	6.72 (0.62)	6.83 (0.43)	6.72 (0.67)	6.78 (0.43)	6.78 (0.59)	12.17
**MODEL 3: PREDICTORS—HBM CONSTRUCTS**						
Susceptibility	10.5 (3.55)	12.25 (3.61)	12.69 (3.36)	13.26 (3.43)	13.45 (4.56)	95.39
Severity	11.39 (4.29)	13.6 (4.04)	14.64 (3.52)	15.75 (3.52)	16.32 (3.76)	150.81^**^
Benefits	5.8 (2.6)	7.69 (2.43)	9.43 (2.34)	10.94 (2.04)	12.77 (2.26)	348.13^**^
Barriers	11.22 (3.7)	10.98 (3.46)	11 (2.76)	9.36 (2.68)	7.82 (3.12)	141.92^**^
Cues to action	4.87 (2.84)	7.19 (2.62)	9.36 (2.32)	10.24 (2.12)	10.76 (2.75)	313.56^**^
**MODEL 4: PREDICTOR—HEALTH ANXIETY**						
Health anxiety	9.72 (8.02)	11.98 (7.16)	13.07 (6.37)	12.46 (6.73)	13.59 (8.05)	172.7

* *p < 0.05;*

** *p < 0.001.*

a *Category variables;*

b *continuous variables*.

The next step involved the effecting of multivariate logistical regressions to test three models.

The first model took as the predictor variables flu vaccination data from the previous season and general vaccination history and as the criterion variable vaccination intention. According to the χ^2^ index, this model is statistically significant [$\chi_{\left(8\right)}^{2}$ = 81.38; *p* < 0.001]. The model correctly predicts 34.7% of responses and the Cox and Snell *R*^2^ is 17%. In this model only general vaccination history was a significant predictor (*p* < 0.001).

The second model took as predictor variables resources (the vaccine being free, vaccine accessibility, and experience of COVID-19 among social circle) and as the criterion variable vaccination intention. Here too the model was statistically significant [$\chi_{\left(12\right)}^{2}$ = 237.69; *p* < 0.001]. 43.1% of responses were correctly predicted by this model and the Cox and Snell *R*^2^ indicator is 42%. For this model the significant predictors were the vaccine being free and its accessibility.

The third model took as predictor variables beliefs connected with health, in accordance with the HBM model, and as the criterion variable vaccination intention. This model was statistically significant [$\chi_{\left(16\right)}^{2}$ = 403.317; *p* < 0.001] and correctly predicted 53.9% of responses. The Cox and Snell *R*^2^ indicator is 60%. Severity, benefits, barriers, and cues to action were all significant predictors for this model.

These results are summarized in Table 4.

**Table 4:** Multinomial logistical regressions.

	**Definitely not OR**	**95%CI**	**Probably not OR**	**95%CI**	**Undecided OR**	**95%CI**	**Probably OR**	**95%CI**
**MODEL 1: VACCINATION HISTORY**								
Vaccinated against flu in previous season	0.18	(0.02, 1.48)	0.58	(0.19, 1.77)	0.71	(0.34, 1.50)	0.48	(0.22, 1.06)
Accepted all previous vaccinations	0.05^**^	(0.02, 0.15)	0.18.58^**^	(0.08, 0.42)	0.36^**^	(0.18, 0.71)	0.97	(0.47, 2.02)
Chi-square (df)	81.38							
% Correct predictions	34.7%							
Cox and Snell *R*^2^	0.17							
**MODEL 2: RESOURCES**								
Experience of COVID-19 among social circle	0.52^**^	(0.32, 0.85)	0.79	(0.51, 1.22)	0.77	(0.55, 1.08)	0.91	(0.66, 1.24)
Encouraged by the vaccine being free	0.002^**^	(0.00, 0.01)	0.01^**^	(0.00, 0.04)	0.02^**^	(0.00, 0.07)	0.20^**^	(0.07, 0.57)
Encouraged by the accessibility of the vaccine	0.13	(0.13, 1.29)	1.15	(0.48, 2.79)	3.23^**^	(1.56, 6.70)	1.76	(0.93, 3.32)
Chi-square (df)	237.69							
% Correct predictions	43.1%							
Cox and Snell *R*^2^	0.42							
**MODEL 3: PREDICTORS—HBM CONSTRUCTS**								
Severity	0.73^**^	(0.62, 0.86)	0.80^**^	(0.69, 0.92)	0.80^**^	(0.71, 0.91)	0.89	(0.80, 1.00)
Benefits	0.35^**^	(0.26, 0.46)	0.40^**^	(0.31, 0.51)	0.50^**^	(0.41, 0.61)	0.68^**^	(0.57, 0.81)
Barriers	2.19^**^	(1.79, 2.69)	2.03^**^	(1.68, 2.46)	1.84^**^	(1.56, 2.17)	1.27^**^	(1.12, 1.45)
Cues to action	0.52^**^	(0.40, 0.66)	0.69^**^	(0.56, 0.85)	0.90	(0.76, 1.08)	1.04	(0.90, 1.21)
Chi-square (df)	403.317							
% Correct predictions	53.9%							
Cox and Snell *R*^2^	0.60							

** *p < 0.001*.

For the significant predictors in the HBM model, supplementary logistical regressions were performed. These showed which of the items included had a significant influence on vaccination intention (Table 5).

**Table 5:** Multinomial logistical regressions – individual items of HBM as predictors.

	**Definitely not OR**	**95%CI**	**Probably not OR**	**95%CI**	**Undecided OR**	**95%CI**	**Probably OR**	**95%CI**
**SEVERITY**								
The disease caused by this virus is more serious than other diseases	0.65	(0.40, 1.06)	0.76	(0.49, 1.18)	0.76	(0.54, 1.07)	1.02	(0.72, 1.45)
The problems I would face from this disease would last a long time	0.93	(0.57, 1.51)	0.94	(0.61, 1.46)	0.95	(0.67, 1.33)	0.93	(0.66, 1.30)
My educational/professional activity would be seriously affected if I were to have this disease	0.79	(0.53, 1.17)	0.88	(0.61, 1.27)	0.80	(0.61, 1.06)	0.94	(0.71, 1.25)
If I had this disease my family's health would be endangered	0.57^*^	(0.38, 0.85)	0.59^*^	(0.40, 0.88)	0.71^*^	(0.51, 0.99)	0.77	(0.55, 1.08)
The mortality rate from this disease is a high one.	0.58^*^	(0.38, 0.87)	0.93	(0.65, 1.35)	1.23	(0.92, 1.65)	1.11	(0.82, 1.49)
Chi-square (df)	78.25 (20)							
% Correct predictions	38.9%							
Cox and Snell *R*^2^	16%							
**BENEFITS**								
My being vaccinated will reduce the probability of my developing COVID-19	0.07^**^	(0.02, 0.18)	0.12^**^	(0.05, 0.29)	0.19^**^	(0.09, 0.40)	0.51	(0.26, 1.01)
My being vaccinated will reduce the probability that members of my family will become ill	0.61	(0.24, 1.57)	0.60	(0.25, 1.42)	0.90	(0.44, 1.82)	0.57	(0.30, 1.10)
My being vaccinated will allow me to resume some of my pre-pandemic activities	0.42^*^	(0.24, 0.73)	0.60^*^	(0.37, 0.98)	0.59^*^	(0.41, 0.84)	0.89	(0.63, 1.27)
Chi-square (df)	271.843 (12)							
% Correct predictions	53%							
Cox and Snell *R*^2^	46%							
**BARRIERS**								
I am worried about the effectiveness of the vaccine	1.35	(0.79, 2.32)	3.34^**^	(1.82, 6.12)	1.66^*^	(1.07, 2.57)	1.15	(0.75, 1.77)
I am worried about possible side-effects of the vaccine	1.84^*^	(1.06, 3.21)	1.03	(0.60, 1.77)	1.63^*^	(1.07, 2.49)	1.32	(0.87, 1.99)
I am worried about whether the vaccine will be distributed and administered correctly	1.15	(0.69, 1.92)	0.78	(0.47, 1.29)	0.97	(0.65, 1.54)	1.02	(0.69, 1.51)
Chi-square (df)	83.00 (12)							
% Correct predictions	41%							
Cox and Snell *R*^2^	17%							
**CUES TO ACTION**								
I will be vaccinated if public figures I trust are vaccinated in public	0.93	(0.52, 1.62)	1.29	(0.84, 1.97)	1.14	(0.85, 1.52)	1.00	(0.71, 1.31)
I will be vaccinated if my family doctor encourages me to do so	0.39^*^	(0.19, 0.80)	0.31^**^	(0.19, 0.50)	0.59^*^	(0.42, 0.83)	0.81	(0.58, 1.13)
I will be vaccinated if I am given appropriate information about the vaccine	0.20^**^	(0.11, 0.37)	0.49^**^	(0.32, 0.75)	0.70	(0.48, 1.02)	0.91	(0.61, 1.34)
Chi-square (df)	204.671^**^ (12)							
% Correct predictions	41.9%							
Cox and Snell *R*^2^	37.7%							

** *p < 0.001;*

* *p < 0.01*.

## Discussions

This study was designed to analyze the way in which vaccination history, resources, HBM constructs and health anxiety influence vaccination intention. The findings partially uphold our research hypotheses. The factors that have a significant influence on vaccination intention were found to be general vaccination history, the freeness and accessibility of the vaccine, severity, the perceived benefits of the vaccine, barriers to vaccination, and cues to action. It became clear that health anxiety is not a predictor of accepting the COVID-19 vaccine.

The first observation to be made refers to the structure of the sample that was included in the study. The fact that most of the participants were young (81.7%), that those of adult-middle age accounted for a percentage of 16.4% and seniors accounted for only 1.9%, may impact the degree of generalization of the results.

Under the influence of factors mentioned above, 45.37% of participants in the study displayed a positive attitude to the vaccine, 22.69% had a negative attitude and the remaining 31.94% stated that they had not yet made up their minds. In the subgroup of middle-aged adults, the attitude toward the vaccine was more favorable than in the group of young people (59.2 and 42% respectively) and there were fewer undecided people (28.2% compared to 32.9%). These differences can be explained by the fact that in general young people are in good health and perceive a lower risk of developing severe forms of the disease. The level of vaccine acceptance among the sample investigated in this study is lower than that found in other population groups. The research carried out by Pogue et al. (2020) in the US found a support rate of 68%, that by Peretti-Watel et al. (2020) in France an acceptance rate of 74%, and in China Wang et al. (2020) observed a vaccine acceptance rate of 91%. These results show that vaccine acceptance levels differ significantly, in some cases being in the region of the 67% threshold that provides herd immunity, which could make it questionable whether it can be attained.

Analyzing vaccination intention in relation to demographic data shows that the categories most prone to refuse the vaccine are young people, men, those in a relationship, and those living in the rural environment. Again, looking at the distribution of those who do not have a definite opinion about vaccination, it was found that the percentage of undecided people was greater among young people, women, single people, those living in the rural environment, those professionally inactive, and those without chronic conditions. These data demonstrate the existence of groups within the population which should be targeted with educational campaigns designed to increase the acceptability of the COVID-19 vaccine. In this context, adult education should be a priority, as this could contribute to the change of perception related to vaccination as a way to increase the level of wellbeing (Goian, 2014). An adequate language used by those involved in the educational process is crucial in obtaining the results we need, by using a to specialized medical language people might get even more scared and could understand even less the necessity of the vaccination and withdraw from the program (Goian, 2010, 2012).

Results obtained in this research study have shown that the largest proportion of correct responses (53.9%) was predicted by the model in which the predictors were HBM constructs, followed by the model using resources, which correctly predicted 43.1% of responses, and the model in which vaccination history was taken as the predictor (34.7% of correct predictions). Looking at the variation of vaccination intention, it is HBM constructs that have the greatest predictive capacity, followed by resources and vaccination history.

For this sample, perceived barriers have the greatest influence on vaccine acceptability. Of these, the possible inefficacy of the vaccine and its possible side effects had significant effects. These results are in agreement with those obtained by Wong et al. (2020), who showed that possible side effects have a significant influence on vaccine acceptability, and with those reported by Pogue et al. (2020), who showed that perceived vaccine efficacy is significantly associated with its acceptance by a population. A further predictor of vaccination intention is the perceived severity of the consequences of the disease. Other research projects too have highlighted this relationship (Graffigna et al., 2020), and the fact that only 44.67% of participants in our research regard this disease and its consequences as serious suggests that there is a need to increase public perception of its seriousness. Although the perceived benefits of vaccination also had a significant influence on fostering positive attitudes toward the vaccine, only 44.21% of respondents see them as very important, which again leaves room for improvement *via* future initiatives. This significant influence of perceived benefits on vaccination intention was also found by Wong et al. (2020). Another HBM construct that had a significant effect in regard to creating support for the vaccine was cues to action. Of these, family doctors' encouragement of vaccination and the availability of suitable information regarding the vaccine were statistically significant. This result also has a practical application, since it shows that both providing the population with information about the vaccine and encouraging family doctors to make clear their support for it could make a major contribution to persuading those who have not yet formed a definite view about the vaccine to accept it. This result is in harmony with those of Wang et al. (2020), who in their turn found that a doctor's recommendation had a significant influence on the decision to be vaccinated.

Of the various resources available to people that were taken into consideration as possible predictors, only the freeness and accessibility of the vaccine had a significant positive influence on attitudes toward COVID-19 vaccination. This result is of importance in relation to the way vaccination campaigns are organized, especially in countries in which the rural population makes up a significant percentage of the total. In the study by Wang et al. (2020) too, both the cost of the vaccine and its accessibility (convenience of access) had a significant influence on people's decision to be vaccinated.

Other factors analyzed in this research with regard to their potential influence on the decision to be vaccinated were general vaccination history and whether the person had had the flu vaccine the previous season. Of these, only general vaccination history had a significant influence. This result is in agreement with those of previous published research; the importance of vaccination history in relation to COVID-19 vaccination has been brought out in other research studies, including those of Pogue et al. (2020) and Wang et al. (2020).

Another aspect highlighted in this study was that health anxiety is not a predictor of decision to be vaccinated. Previous studies have described the different reactions that people with health anxiety can exhibit during the pandemic: avoiding visiting doctors' offices, seen as potential sources of infection, or the opposite extreme of seeking reassurance by making an excessive number of visits to emergency health care centers whenever they experience bodily sensations that could be interpreted as signs of infection. People with a high degree of health anxiety may also, during a pandemic, withdraw from society, wash their hands to excess, or indulge in panic buying (Asmundson and Taylor, 2020). One possible explanation for the finding that health anxiety does not increase the intention to be vaccinated could be that fear of the vaccine and its possible side effects may be greater than fear of the disease.

Identifying groups within the population which display a low level of vaccine acceptance, and discovering which factors influence the decision to be vaccinated, are both essential to the successful rollout of a vaccination campaign. The importance of this study lies precisely in the fact that it contributes supplementary information regarding these points and that its findings can be used by those designing both educational programmes about the vaccine and a vaccination strategy. Our research results show that a high percentage of respondents (31.94%) have not yet made up their minds. These are the people who should be targeted first by programmes of this kind. According to this study, the psychological triggers to be borne in mind are perception of the seriousness of the disease and of its consequences, together with perception of the benefits of being vaccinated, of barriers, and of cues to action. Particular attention should be paid to disseminating available information about the vaccine among the population and to family doctors expressing their support for vaccination. With respect to resources available at society level, the freeness of the vaccine makes a significant contribution to vaccine acceptance. In addition, to increase vaccination uptake, the campaign should ensure the accessibility of the vaccine from the point of view of vaccination method, frequency, and distance to the place where vaccination is carried out.

Our research also has a number of limitations. In the first place, using as it does a cross-sectional type design, it does not allow the dynamic between different variables to be highlighted, nor can conclusions be drawn regarding causality. We should also draw attention to the fact that the research was carried out online and that a convenience sample was used, which may impact the degree to which general conclusions can be drawn from our findings. Thus, given that most of the people included in the study were young and the demographic variables were not statistically controlled, the results should be interpreted with the utmost caution. Last but not least, it must be noted that the data were self-reported, which may expose them to reporting bias.

With regard to future research projects, as vaccination campaigns move forward these will be able to take into account actual vaccination behaviors and not merely expressed intentions. Additionally, it would be possible to analyze the relationship between other psychological variables, such as personality traits, and willingness to be vaccinated. Another category of studies that might be devised in the future would be aimed at identifying the factors that influence vaccination acceptance and vaccination behavior among vulnerable people, such as those with comorbidities and those aged 65 and over.

## Conclusion

The HBM constructs—severity, benefits, barriers, and cues to action—are significant predictors of vaccination intention. To these may be added the freeness and accessibility of the vaccine and general vaccination history. The results of this study can be used in interventions that aim to improve attitudes toward COVID-19 vaccination, but they should be interpreted with caution because the study used a sample of convenience in which most participants were young and the demographic variables were not statistically controlled.

## Data Availability Statement

The original contributions presented in the study are included in the article/supplementary material, further inquiries can be directed to the corresponding author/s.

## Ethics Statement

This study was approved by the Ethics Committee of West University of Timişoara (decision no. 5224/ 03.02.2021). All participants expressed informed consent.

## Author Contributions

All authors listed have made a substantial, direct and intellectual contribution to the work, and approved it for publication.

## Conflict of Interest

The authors declare that the research was conducted in the absence of any commercial or financial relationships that could be construed as a potential conflict of interest.

## Publisher's Note

All claims expressed in this article are solely those of the authors and do not necessarily represent those of their affiliated organizations, or those of the publisher, the editors and the reviewers. Any product that may be evaluated in this article, or claim that may be made by its manufacturer, is not guaranteed or endorsed by the publisher.

## Footnotes

^1^Ştefănuţ, A. M., and Vintilă, M. (submitted). The influence of demographic features, experience of the pandemic, and beliefs about health on behaviours aimed at preventing COVID-19. Manuscript submitted for publication.
